# Conditional generative adversarial network driven radiomic prediction of mutation status based on magnetic resonance imaging of breast cancer

**DOI:** 10.1186/s12967-024-05018-9

**Published:** 2024-03-02

**Authors:** Zi Huai Huang, Lianghong Chen, Yan Sun, Qian Liu, Pingzhao Hu

**Affiliations:** 1 Department of Biochemistry, Schulich School of Medicine & Dentistry, Western University, London, ON Canada; 2 Department of Computer Science, Western University, London, ON Canada; 3 Department of Applied Computer Science, University of Winnipeg, CH Room 3C08B, 515 Portage Avenue, Winnipeg, MB R3B 2E9 Canada; 4 Department of Epidemiology and Biostatistics, Schulich School of Medicine & Dentistry, Western University, London, ON Canada; 5 Department of Oncology, Schulich School of Medicine & Dentistry, Western University, London, ON Canada; 6 The Children’s Health Research Institute, Lawson Health Research Institute, London, ON Canada; 7 Department of Biochemistry, Western University, Siebens Drake Research Institute, SDRI Room 201-203B, 1400 Western Road, London, ON N6G 2V4 Canada

**Keywords:** Breast cancer, cGANs, Radiogenomics, Machine learning, Magnetic resonance images

## Abstract

**Background:**

Breast Cancer (BC) is a highly heterogeneous and complex disease. Personalized treatment options require the integration of multi-omic data and consideration of phenotypic variability. Radiogenomics aims to merge medical images with genomic measurements but encounter challenges due to unpaired data consisting of imaging, genomic, or clinical outcome data. In this study, we propose the utilization of a well-trained conditional generative adversarial network (cGAN) to address the unpaired data issue in radiogenomic analysis of BC. The generated images will then be used to predict the mutations status of key driver genes and BC subtypes.

**Methods:**

We integrated the paired MRI and multi-omic (mRNA gene expression, DNA methylation, and copy number variation) profiles of 61 BC patients from The Cancer Imaging Archive (TCIA) and The Cancer Genome Atlas (TCGA). To facilitate this integration, we employed a Bayesian Tensor Factorization approach to factorize the multi-omic data into 17 latent features. Subsequently, a cGAN model was trained based on the matched side-view patient MRIs and their corresponding latent features to predict MRIs for BC patients who lack MRIs. Model performance was evaluated by calculating the distance between real and generated images using the Fréchet Inception Distance (FID) metric. BC subtype and mutation status of driver genes were obtained from the cBioPortal platform, where 3 genes were selected based on the number of mutated patients. A convolutional neural network (CNN) was constructed and trained using the generated MRIs for mutation status prediction. Receiver operating characteristic area under curve (ROC-AUC) and precision-recall area under curve (PR-AUC) were used to evaluate the performance of the CNN models for mutation status prediction. Precision, recall and F1 score were used to evaluate the performance of the CNN model in subtype classification.

**Results:**

The FID of the images from the well-trained cGAN model based on the test set is 1.31. The CNN for *TP53, PIK3CA,* and *CDH1* mutation prediction yielded ROC-AUC values 0.9508, 0.7515, and 0.8136 and PR-AUC are 0.9009, 0.7184, and 0.5007, respectively for the three genes. Multi-class subtype prediction achieved precision, recall and F1 scores of 0.8444, 0.8435 and 0.8336 respectively. The source code and related data implemented the algorithms can be found in the project GitHub at https://github.com/mattthuang/BC_RadiogenomicGAN.

**Conclusion:**

Our study establishes cGAN as a viable tool for generating synthetic BC MRIs for mutation status prediction and subtype classification to better characterize the heterogeneity of BC in patients. The synthetic images also have the potential to significantly augment existing MRI data and circumvent issues surrounding data sharing and patient privacy for future BC machine learning studies.

**Supplementary Information:**

The online version contains supplementary material available at 10.1186/s12967-024-05018-9.

## Introduction

Breast cancer (BC) is currently the tumor with the highest incidence rate worldwide and has just surpassed lung cancer as the most diagnosed cancer in the world. Its incidence rate in the year of 2020 accounted for 11.7% of all forms of cancer with a total of 2.3 million new cases [1]. Although there have been advancements in personalized treatment options, survival rate for BC has only improved slightly. It is still estimated that from the years 2015–2030, death due to the cancer will increase by 43% [2]. This pattern in BC prognosis is due to the known heterogeneity among breast tumors, which must be addressed in order to better categorize BC patients [1]. Novel machine learning approaches may address the issue of BC heterogeneity, but its validity must be explored [3, 4]. BC heterogeneity can exist either between different patients with the same tumor type (intertumor heterogeneity) or within the same patient (intratumor heterogeneity) [5]. Intratumor heterogeneity can further be differentiated into spatial and temporal heterogeneity. Spatial heterogeneity refers to differences that presents itself in different geographical regions of a tumor while temporal heterogeneity is considered as molecular evolutions of a tumor over time [6]. As a result of these differences among tumors, it poses as a major concern for the development of therapeutic approaches. Intertumor heterogeneity suggests that every BC can be different in every patient; thus, precluding the possibilities of a “one size fit all” treatment [7]. A bigger challenge lies in intratumor heterogeneity which suggests that some drug treatments may not be effective against the whole tumour. The multiple subclones with varying sets of molecular aberrations combined with different drug sensitivities greatly impacts the treatment effectiveness. Furthermore, tumor evolution is in part responsible for differential sensitivity and thus exacerbating the challenge of developing an effective BC treatment [7].

Current treatment options for BC are developed based on a screening/diagnosis procedure known as needle biopsy [8]. Core needle biopsy is the preferred method for screening compared to other methods such as fine-needle aspiration cytology or surgical excision. The tissue obtained from the core needle biopsy provides crucial information regarding tumour type, grade, and the expression of biomarkers. As a result, subsequent analysis and measurements of these biomarkers is crucial in helping guide therapy and providing predictive and prognostic information. More specifically, the molecular characterization of a tumour can integrate information from all different omics profiles. This includes data on changes of genes (genomic profile), mRNA (transcriptomic profile), non-coding RNAs and DNA modifications (epigenomic profile), metabolism (metabolomic profile) and proteins (proteomic profile) [9]. The integration of information from these varying sources can help identify genetic aberrations that allow clinicians to provide the patient with the best therapeutic options. In BC currently, tumours are classified into five different groups: luminal A, luminal B, ErbB2/Her2+ , basal and normal like [10]. Each subtype is marked by its own unique marker expression and is associated with a different prognosis [11]. As a result, proper subtyping, and molecular profile characterization of specific biomarkers such as estrogen receptor (ER), progesterone receptor (PR) and human epidermal growth factor 2 (HER2) is crucial in the creation of personalized treatment options for patients and progression of the disease. However, biopsies of these small tumor regions may not be representative of the entire tumour due to the high heterogeneity that is present with BC. In particular, the genetic, epigenetic, and phenotypic alterations of the entire tumor may not be accurately represented by sampling such a limited area. These imprecisions can lead to under diagnosis of lethal, life-threatening cancers while over diagnosing and over-treating indolent forms of BC [12].

Provided with these limitations associated with performing biopsies, it creates a strong demand for non-invasive and more accurate means of identifying molecular subtypes for BC tumours, such as medical imaging methods or the “omics” field known as radiomics. This acts as a potential alternative for the identification of biomarker mutation statuses [13]. When compared to biopsies, medical imaging provides a full and unbiased view of the tumour without the need for costly, time consuming and invasive procedures. However, it fails to provide the underlying molecular profiles of these biomarkers of interest. With the recent technological advancements, imaging technologies have improved significantly. One of the major advances provides the possibility of spatially examining entire tumours over time both in vivo and non-invasively [13, 14]. Combined with powerful informatics resources, contents that are hidden to the naked eye can be extracted as quantitative features from the images of the tumour [15].

This new paradigm in the field of radiomics integrated with genomic information opens the doors to the novel field of radiogenomics and allows for unprecedented insight into the complex tumor biology of BC [12]. Radiogenomics is based on the idea that biomedical images reflect genetic and molecular processes. Therefore, imaging parameters derived from advanced image processing and analysis can provide insight into the underlying molecular and genotypic makeup of tissues, addressing the flaw in the utilization of radiomics [15]. In a pilot study conducted in 2012, a radiogenomics association map linking magnetic resonance image (MRI) phenotype to underlying global gene expression patterns in BC was created [16, 17]. Several correlations were identified between imaging traits and genes measured in the BC patients and demonstrated promising results as evidence for the field of radiogenomics. The growing literature in this field relies exclusively on MRI or more specifically, dynamic contrast material-enhanced MRI (DCE-MRI) [18, 19]. However, one major problem that is distinct to BC and radiogenomics studies is still currently present in the form of unpaired data.

In order to conduct a thorough radiogenomics study, three different data types are needed. Multi-omics data for the tumours underlying molecular profile, imaging data for feature extraction and the patient’s clinical outcome are all needed for correlational or causal conclusions to be drawn. Currently in the field, unpaired data is present where one dataset may contain medical images and genomics data for the same patient, but the patient’s clinical outcome is missing. For instance, a dataset may include both medical images and genomics data for the same patient, which allows for feature extraction and radiogenomics mapping [9]. However, it is often difficult to obtain clinical outcomes for these same patients as it requires long-term observations. As a result, the ability of the image features to predict outcomes, known as their prognostic significance, cannot be evaluated and they cannot be identified as a prognostic biomarker. To address the unpaired data problem, recent studies have examined the potential use of deep learning approaches in the generation of medical images for those patients with genomic and clinical information.

Deep learning is a subset of machine learning algorithms that utilize artificial neural networks (ANN), which are inspired by the structure and function of the human brain [20, 21]. These neural networks can be combined and configured in a way to perform image classification tasks and even the synthesis of medical images as well. One model in particular, known as a conditional generative adversarial network (cGAN) has shown great potential [22–26]. Currently, GANs have been used for BC lesion detection and subtype classification [27]. However, the model can also be used to generate synthetic images that do not contain any real patient data. This ability will be useful for training and testing other related machine learning models, as it allows for the creation of larger datasets without the need to acquire additional real patient images [22, 28]. More importantly, the cGAN model’s ability to generate artificial BC MRIs for those patients without imaging will help address the unpaired data problem present in current BC datasets.

Another strength and application of deep learning computer models is its ability to perform image classification tasks. For instance, convolutional neural networks (CNNs) are a type of ANN that is specifically designed for image processing [12]. They are particularly effective for analyzing medial images, as they are able to automatically learn features and patterns in the image that are relevant for diagnosis and treatment [29]. One of the key advantages of CNNs for image classification is their ability to automatically learn features from the data, rather than requiring manual feature engineering [22]. This allows the network to learn complex patterns and relationship in the data that may not be immediately apparent to human analysts. CNNs has been supported by previous studies in its ability to predict ER status better than traditional immunohistochemistry stains [29].

The study of BC heavily relies on genomic data, specifically copy number variation (CNV), gene expression, and DNA methylation. These types of data can reveal genetic alterations, molecular subtypes, and epigenetic modifications that have important implications for diagnosis and treatment decisions. Unfortunately, there is currently a lack of matching DCE-MRI data for patients within the various BC databases, which hinders radiogenomic studies in the field.

To address the unpaired data problem, we hypothesize a well-trained cGAN to generate synthetic BC MRIs based on patients’ multi-omic features. The generated images can then be used to predict BC subtypes and the mutation status of key BC driver genes. To prove the proposed hypothesis, three specific aims have been established. 1) Address the unpaired data problem through the generation of synthetic BC MRIs using patients’ multi-omic profiles through a well-trained cGAN; 2) the collection BC subtypes and key BC driver genes and their associated mutation status; and 3) the prediction of BC subtypes and mutation status of the BC driver genes using a CNN based on the generated synthetic images. Through these aims, the study seeks to demonstrate the feasibility and potential utility of cGANs in the field of radiogenomics with regards to BC research.

## Material and methods

### BC dataset

**Fig. 1:**
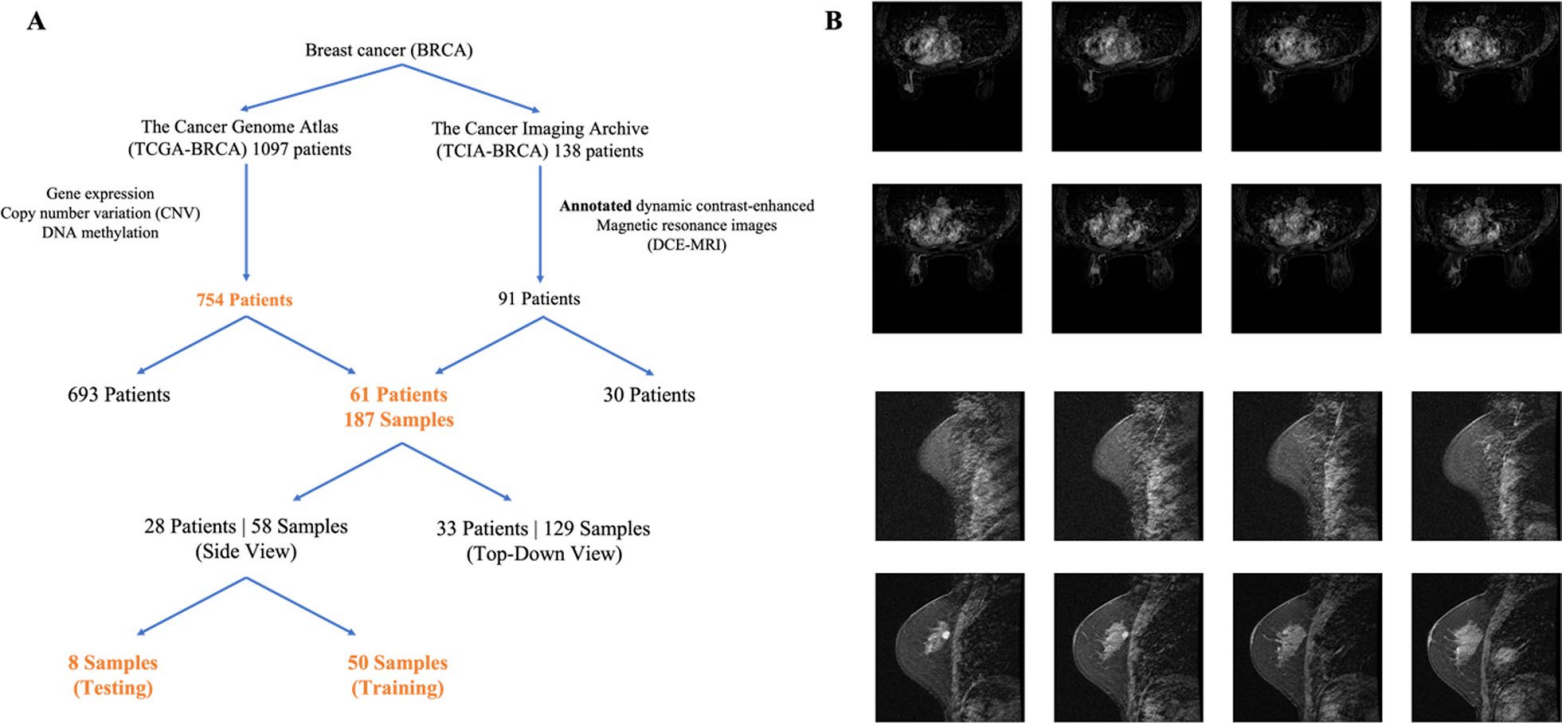
Depiction and visualization of dataset used in study. **A** 754 out of 1097 patients with gene expression, CNV, DNA methylation data were obtained from the TCGA-BRCA cohort. 91 out of 138 patients with annotated DCE-MRI patients were selected from the TCIA-BRCA cohort. Out of the two cohorts, 61 patients contain annotated DCE-MRI, gene expression, CNV, and DNA methylation. The 61 patients with 187 MRI samples are then matched and separated into side view MRIs and top-down view MRIs. Side view MRIs were chosen for use and were further separated into training and testing sets. **B** 2-dimensional visualization of patient MRI. MRI from 61 patient with both genomic data and imaging data. 3-dimensional image is sliced into 32 different slices for visualization purposes. 8 representative slides are shown here. Top-down view MRIs are displayed on top, and side view MRIs are displayed on the bottom

The data that was used in the study consisted of multi-omics data and medical imaging data, each obtained from their respective database as illustrated in Fig. 1A. Multi-omic data consisted of three varying types of genomic data (CNV score, gene expression, DNA methylation) and was retrieved from the Breast Invasive Carcinoma (BRCA) project in The Cancer Genome Atlas (TCGA) platform. After matching, there were 754 patients with all three omics data and a multi-omics tensor was constructed from these three sources. The tensor was then decomposed using the Bayesian Tensor Factorization (BTF) algorithm to generate a patient directional tensor with patients by 17 latent features. It should be noted that BTF multi-omics tensor extraction of the 17 latent multi-omics features have been performed in our previous study [30].

The DCE-MRIs of these BC patients were obtained from The Cancer Imaging Archive (TCIA) [31, 32]. Among the 754 patients in the TCGA-BRCA cohort with genomic information, only 61 have matched DCE-MRI data available. This highlights the aforementioned unpaired data problem in BC databases, where a substantial amount of genomic data is available but there are limited MRIs. The original DCE-MRI data is in Digital Imaging and Communications in Medicine (DICOM) format with rich information about the acquisition settings. We only extracted the digital image pixel values for analysis in the study. There were 187 three-dimensional DCE-MRIs for the 61 patients, meaning that a patient may have multiple three-dimensional DCE-MRIs. These three-dimensional images were acquired at different time points with an interval of dozens of seconds for capturing the dynamic information. To make the three-dimensional images comparable, we resized them into a 32 × 128 × 128 structure. A visualization of the three-dimensional DCE-MRI can be found in Fig. 1B. The available 187 DCE-MRIs for the 61 patients were split into two different views, a side view, and a top-down view of the breast, as shown in Fig. 1B. Of these, 58 samples were side view while 129 samples were top-down view. Although the number of top-down view DCE-MRIs is significantly higher, side view DCE-MRIs were ultimately chosen for training and testing of the model. Side view DCE-MRIs of the breast facilitate easier visual inspections and assessments of the quality of the synthetically produced MRIs. They provide a general shape of the breast that can be assessed by the human eye, which is difficult to do for top-down view DCE-MRIs. The 58 samples are then divided into training and testing sets consisting of 50 images and 8 images respectively (Fig. 1A).

### Study design

**Fig. 2:**
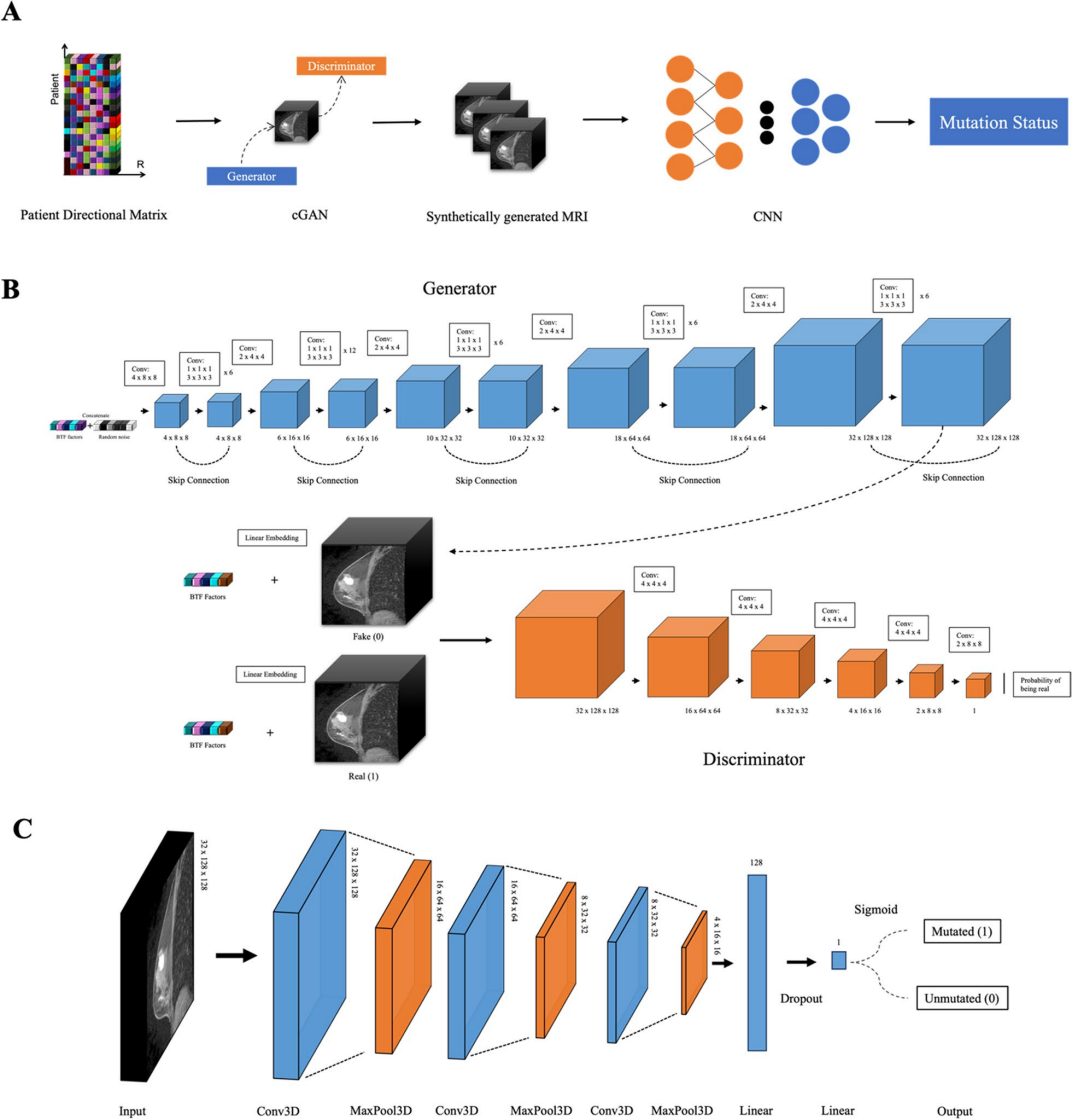
Architecture diagram for deep learning models. **A** Overall study design. Patient directional tensor containing 17 latent features fed into the cGAN for MRI generation and subsequent mutation status prediction. **B** Architecture diagram of cGAN model. **C** Labelled synthetic image from cGAN and real patient MRI from TCIA goes through several layers for a binary classification result to be generated

The overall project workflow is shown in Fig. 2A. A multi-omic tensor is constructed from three varying sources of molecular information, including gene expression data, CNV score and DNA methylation. The tensor is then decomposed using the BTF algorithm to generate a patient directional tensor with patients by 17 latent features to use as the conditional input for the cGAN. The synthetically produced BC MRIs act as inputs to the CNN for mutation status predictions for BC driver genes of interest and BC subtypes. It should be noted that BTF multi-omic tensor extraction of the 17 latent multi-omic features have been performed in our previous study [30].

### Architecture of cGAN and its evaluation

#### Architecture of cGAN

The cGAN model utilized in this study is based on Ian goodfellow’s 2014 work, which introduced the concept of a GAN comprising of two CNNs: the generator and the discriminator [33]. These networks can be likened to players engaged in a game. The generator network’s objective is to produce synthetic data, such as artificial MRIs, that are highly similar to real images, while the discriminator network aims to distinguish between real and generated data. As the game progresses, the generator enhances its ability to differentiate between real and synthetic data. Back propagation, Markov chains, and dropout techniques are employed by both networks to facilitate learning and mutual improvements [34]. Ultimately, the generator aims to generate an image that is virtually indistinguishable from a real image, rendering the discriminator incapable of differentiating between the two. In this study, we added a conditional factor input for the generator that is derived from the latent multi-omic features obtained from the BTF for these 50 side view patients. The generated images are then evaluated by the discriminator to determine the probability of authenticity, as illustrated in Fig. 2B.

#### cGAN model training and evaluation

Training of the cGAN involved several key hyperparameters as outlined in Table 1. Different iterations of the hyperparameters were tested and compared by computing a Fréchet Inception distance (FID) score for the models. FID score measures the distance between the distribution of the real images and the distribution of the synthetic images, in terms of the features learned by a pre-trained inception model. A lower FID score indicates more similarities between the real and fake images which in turn represents better cGAN performance. Implementation of the FID score was altered to accommodate for the three-dimensional nature of the MRIs obtained from TCIA dataset. Instead of using the traditional Inception V3 network to extract features, a pretrained 3D CNN called Med3D is used [35, 36]. The Med3D network is specifically designed for medical image analysis tasks and has shown to outperform general—purpose CNNs such as Inception V3 on several medical image datasets. Med3D utilizes 3D convolutional layers to capture the spatial information present in 3D medical images [35]. The batch size parameter and epoch parameter were limited by the computing power of the machine as higher batch sizes demanded more system memory and higher number of training epochs led to significantly longer training durations. The model was also validated using ten-fold cross validation to minimize possible bias form the train-test split and to provide a more robust representation of model performance. The model was trained on a NVIDIA 1660 Ti with 16 GB of RAM with the bolded hyper-parameters in Table 1.

**Table 1:** cGAN hyperparameters. Parameters that were used for training are outlined in the table. Bolded specifications represent the parameters that were used to train the final model

Hyper-parameters	Specifications
Batch size	1, **2**, 5, 10
Generator learning rate	0.0000112, **0.000025**, 0.00175, 0.0025
Discriminator learning rate	0.0000045,0.0000075,**0.00001**, 0.0001
Loss	**MSELoss,** BCELoss, L1Loss
Epochs	50, 150, 250, 300, 750, 880, **1200**, 1500
Leak value	**0.2**

### Clinical applications of cGAN MRIs

#### Mutation status prediction of key BC driving genes

A list of key cancer driving genes were obtained from a recent study performed by Bailey et al., where a comprehensive characterization of 299 cancer driver genes were performed [37]. The following three genes were selected for their mutation status prediction based on its role in BC and the number of mutated patients within the dataset: *TP53*, *PIK3CA*, and *CDH1.* The mutation status of these BC driver genes were obtained from the Breast Invasive Carcinoma (TCGA, PanCancer Atlas) dataset at the cBioPortal platform [38, 39]. The mutation status for each gene in each BC patient is displayed either as a 0 for an unaltered status or a 1 for an altered status.

#### BC subtype prediction

To further evaluate the clinical applicability of cGAN generated MRIs, clinical data available on the cBioPortal platform were collected [38, 39]. This includes five BC subtypes Basal, Normal, LumA, LumB and HER2. To facilitate downstream multiclass classification, these subtypes were mapped to integer values as follows: Normal (0), Basal (1), LumA (2), LumB (3), and HER2 (4).

### Architecture of CNN and its evaluation

#### Architecture of CNN

The CNN network consists of multiple layers of interconnected “neurons”, which process and analyze the input data. The first layers of a CNN typically consist of convolutional layers, which apply a set of filters to the input image to extract features such as edges and patterns. These filters are learned during the training process and can identify specific features in the data that are important for classification. The constructed CNN model consists of three convolutional layers, with 32, 16, and 8 output channels respectively, each followed by a max-pooling layer [40, 41]. The output of the final pooling layer is then flattened and passed through two fully connected layers with 128 and 1 output neurons respectively. The model also includes a dropout layer with dropout probability of 0.5 to prevent overfitting. The activation function used throughout the network is the rectified linear unit (ReLU), except for the output layer which uses the sigmoid function to produce a binary classification output. This is appropriate as the mutation status for the various genes are labeled in binary as either “0” or “1”. To perform subtype classification, the sigmoid function will be removed to perform multi-class classification. A summary of the model structure for mutations status prediction is shown in Fig. 2C.

#### CNN model training and evaluation for mutation status predictions

The CNN was trained with both real patient MRI and cGAN produced MRIs to compare results and to assess the predictive power of the synthetically generated MRIs. The genes of interest *TP53*, *PIK3CA* and *CDH1* had the greatest number of mutated patients and were therefore chosen for mutation status prediction. Although the well-trained cGAN produced a total of 754 synthetic MRIs, only 690 of these matched to the mutation status of the three genes. The number of mutated patients in both real and cGAN MRI datasets are outlined in Table 3. Test sets for both consists of 20 percent of the total number of patients. These datasets were then used as inputs for the CNN to complete training using parameters as outlined in Table 2. CNN using real MRI were trained for 300 epochs for all 3 genes while cGAN CNNs were trained for 1300, 1500, 2000 epochs for *CDH1*, *PIK3CA* and *TP53* respectively.

**Table 2:** CNN hyperparameters. Parameters that were used for training/testing of the CNN. Bolded parameters represents those that were used to train the final model

Hyper-parameters	Specifications
Batch Size	**1**
Optimiser	SGD, **Adam**
Learning Rate	StepLR, CyclicLR, **ReduceLROnPlaeau**
Loss	MSELoss, **CrossEntropyLoss**, **BCELoss,** NLLLoss
Epochs	50, 75, 100**,** 150, 250, **300**, 600, 900, **1300**, **1500**, 1800, **2000**, 2200, **2500**

Predictions that are made from the CNN are evaluated through two performance metrics, a receiver operating characteristic (ROC) curve and a precision and recall (PR) curve. ROC curve is a graphical representation of the performance of a binary classifier system as its discrimination threshold is varied. It plots the true positive rate against the false positive rate for different threshold values. The Area Under the Curve (AUC) of the ROC curve is a widely used performance metric that indicates the overall quality of a classifier system. An AUC of 1 represents a perfect classifier while an AUC of 0.5 would indicate a random guess [29]. The method was chosen for its previous success in similar studies. For instance Han et al. applied the ROC curve and AUC to assess the performance of a machine learning model in identifying BC subtypes based on gene expression data [42].

A PR curve plots the trade-off between precision and recall (sensitively) for different classification thresholds. The AUC for the PR curve is another common metric used to evaluate performance of a model, with a value of 1 indicating a perfect model and a value of 0 representing a random model. PR curves are insensitive to changes in the negative class distribution and therefore perform better for imbalanced datasets such as the datasets at hand [43].

#### CNN model training and evaluation for subtype predictions

The CNN was trained using cGAN generated MRIs and a combined dataset containing both real patient and cGAN generated MRIs. 659 out of 754 cGAN MRIs successfully mapped to a subtype label from the cBioPortal platform. However, in the real patient dataset, only 23 patients had a subtype label. Due to the limited number of labeled data in the real patient dataset, it was excluded from subtype predictions, and the evaluation focused on the cGAN and combined datasets. The CNN was trained for 2500 epochs for both datasets using bolded parameters in Table 2.

For this multi-class classification task, precision, recall, and the F1 score were used as performance metrics. Precision represents the ratio of true positive to the total number of positive predictions (sum of true positives and false positives). A high precision score indicates lower rates of false positive predictions. Recall measures the rate of false negative predictions, with a high score suggesting lower rates of false negatives. It calculates the ratio of true positive predictions to the total number of real positives (sum of true positives and false negatives). The F1 score is the harmonic mean between precision and recall, where a higher score indicates better precision and recall.

## Results

### Performance of the constructed cGAN model

**Fig. 3.**
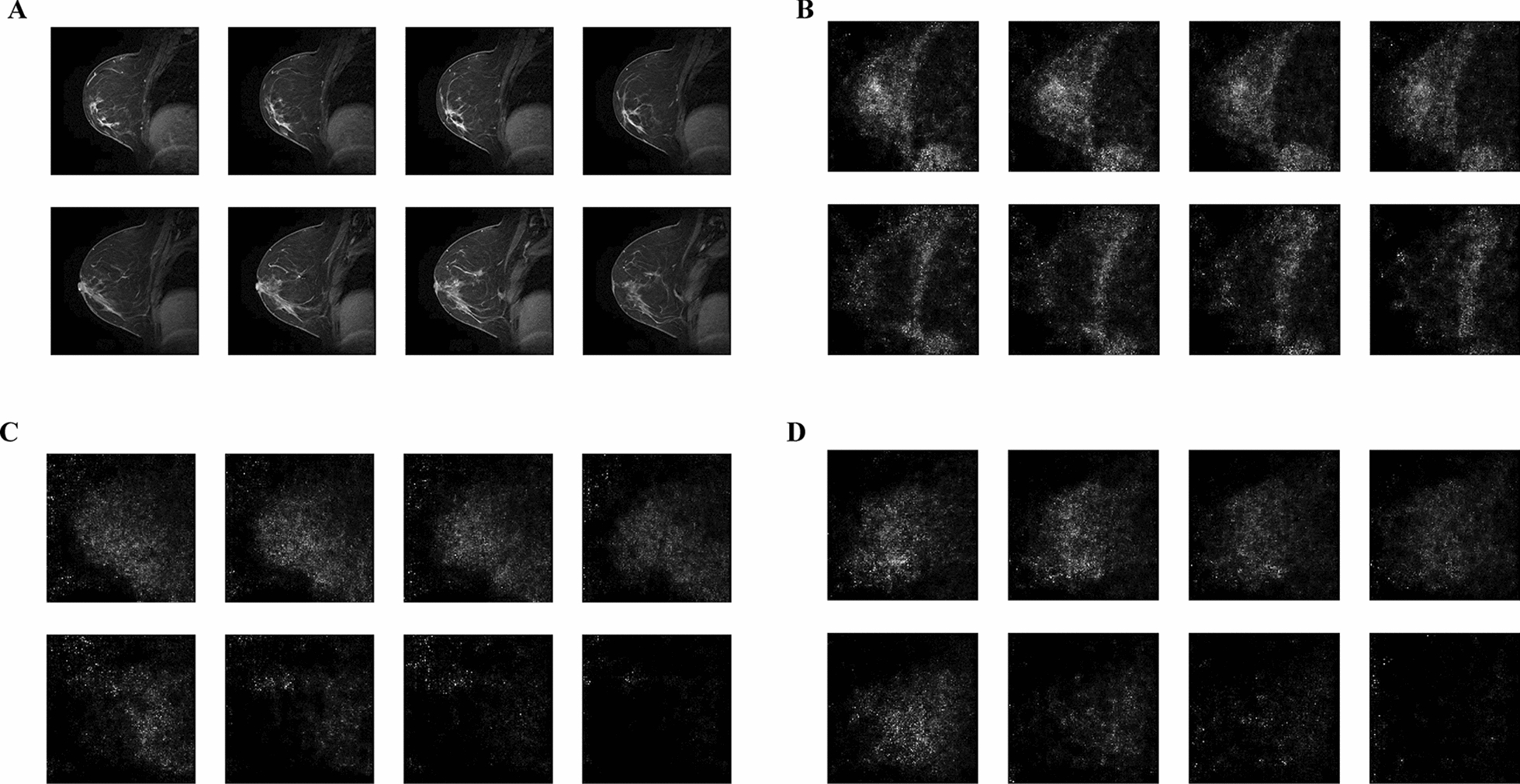
8 slices of 32 total slices of the 3D MRI BC images of patient TCGA-AO-A12E. **A** Original patient MRI visualized in 32 slices. **B** Synthetically generated MRI from well-trained cGAN. **C** Synthetically generated MRI from well-trained autoencoder with Resnet 18. **D** Synthetically generated MRI from well-trained autoencoder

The cGAN model was trained based on the bolded parameters outlined in Table 1. Additional file 1: Fig. S3. depicts the loss curve generated using the mean squared error (MSE) loss function. The loss converges to a single value as the number of epochs increases, indicating that the model is well trained. The original patient MRI is displayed in Fig. 3A, with the final MRIs that were predicted by the cGAN with only the patients 1 × 17 tensor containing their genomic information in Fig. 3B. Figure 3C shows the MRIs generated from a Resnet 18 pretrained autoencoder, and Fig. 3D displays the MRI generated from a traditional autoencoder. These 8 slices are 2-dimensional visualizations of the 3-dimensional MRIs, while the full 32 slices are shown in Additional file 1: Figs. S4–S7.

The final generated MRIs from the test dataset using only genomic information is tested against the real MRIs based on a generated FID score. A traditional auto-encoder and an auto-encoder with a pretrained Resnet 18 were trained and used as a baseline measurement for comparison. All three models were validated using tenfold cross validation with the FID score (Table 3). The images generated from the cGAN yielded a lower FID score across all folds while the conventional autoencoder had the highest. Most notably, all three models had the lowest score in fold 3 with the best being the cGAN at 1.31 ± 0.57. These significant differences indicate the effectiveness of the cGAN model compared to traditional methods. The lowest scoring fold 3 weight was chosen to generate MRIs for patients without imaging data. Its clinical applicability in mutation status prediction and subtype classification will be validated using the CNN model. However, a visual inspection of the generated MRIs with the human eye revealed that while the general breast shape could be observed, the machine-generated images lacked finer details (Fig. 3B).

**Table 3:** FID scores for cGAN and baseline models. All models were evaluated using 10-fold cross validation. FID scores were calculated against the matching real patient MRI. Bolded fold represents the fold that yielded the best FID score for all three models (fold 3)

	cGAN	Pretrained autoencoder	Autoencoder
CV Fold 0	1.51 ± 0.67	3.41 ± 0.82	4.89 ± 0.52
CV Fold 1	1.91 ± 0.57	3.52 ± 0.75	3.90 ± 0.85
CV Fold 2	1.79 ± 0.64	3.32 ± 0.94	3.51 ± 0.92
CV Fold 3	**1.31 ± 0.57**	**3.80 ± 0.73**	**3.76 ± 0.75**
CV Fold 4	3.42 ± 0.72	4.69 ± 0.82	5.21 ± 0.94
CV Fold 5	3.14 ± 0.49	4.57 ± 0.53	5.16 ± 0.53
CV Fold 6	2.97 ± 0.38	4.08 ± 0.63	4.98 ± 0.41
CV Fold 7	3.45 ± 0.59	4.59 ± 0.94	4.94 ± 0.36
CV Fold 8	3.24 ± 0.94	4.21 ± 0.82	4.60 ± 0.48
CV Fold 9	2.76 ± 0.76	3.87 ± 0.91	4.05 ± 0.83
All folds	2.13 ± 0.33	3.79 ± 0.48	4.31 ± 0.53

### Performance of cGAN MRIs in mutation status prediction

**Fig. 4:**
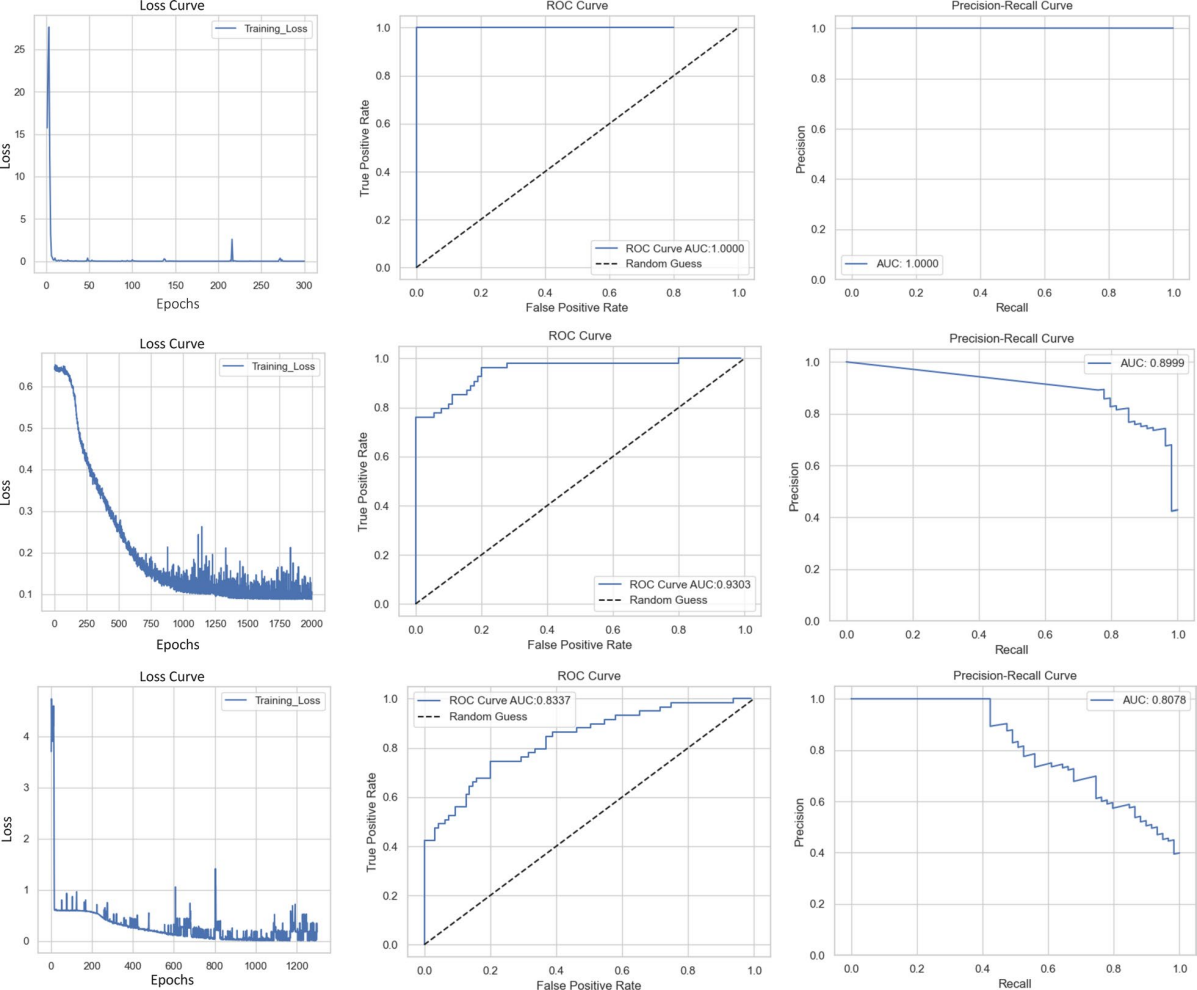
CNN BC loss curve, ROC and PR curves for *TP53*. Top depicts CNN trained using real patient MRIs, middle panel represents CNN trained on cGAN predicted MRIs, and bottom panel for CNN trained with both real and cGAN generated MRIs

Performance of the cGAN MRIs in mutation status prediction were evaluated in two steps with the three genes of interest *TP53, PIK3CA,* and *CDH1.* Three training/testing sets were created with varying proportions of test sets (10, 20 and 30%) to identify the test proportion which would yield the best results. Additional file 1: Table S1 summarizes the ROC and PR AUC values for these varying tests sets that were trained with cGAN produced MRIs. 20% test set for *TP53* and *PIK3CA* yielded the highest AUC values out of the three while *CDH1* observed best values in the 10 percent test set. As 20 percent resulted in the best performance for 2 out of the 3 genes, it was chosen as the proportion of interest for further evaluation. In the next step, the CNN model was trained with real patient MRIs, cGAN produced MRIs, and a combination of real and cGAN produced MRIs, using the 20 percent test set proportion (Table 4**)**. The loss, ROC, and PR curves are presented for *TP53* in Fig. 4., while the curves for *PIK3CA* and *CDH1* are provided as Additional file 1: Figs. S8, S9. A logistic regression with L1 regularization based on pure patient multi-omic data was constructed and evaluated using ROC AUC and PR AUC as a baseline comparison for the CNN results (Additional file 1: Fig. S10). For *TP53*, the logistic regression baseline had a ROC AUC score of 0.9400 and PR AUC of 0.9009. The CNN when trained with real patient images, achieved a perfect AUC value of 1.0000 for both the ROC and PR curves, indicating accurate binary classification of all patients in the test set. The combined dataset of real patient MRIs and cGAN generated MRIs scored the highest AUC scores of 0.9508 for the ROC curve and 0.9301 for the PR curve. *CDH1* reported 0.9167 for ROC AUC and 0.9083 for PR AUC when the CNN was trained with real patient images, and baseline logistic model scored 0.8068 and 0.4342 for ROC AUC and PR AUC respectively. However, the AUC scores for the combined (cGAN and real) dataset remains the second highest among all experiments at 0.8136 and 0.5007 for ROC and PR. *PIK3CA* follows a similar trend where the combined dataset predictions scored just below the dataset with real patient MRIs. The ROC AUC is 0.7515 compared to the higher value of 0.8333 and the PR AUC is 0.7184 compared to 0.8110. Overall, the performance trends of all three genes followed a consistent pattern, with the descending order of ROC AUC scores as follows: real patient MRI, cGAN + real MRI, cGAN MRI, and multi-omic logistic regression.

**Table 4:** ROC AUC and PR AUC scores of a multi-omic based logistic regression and CNN trained with real patient MRIs, cGAN predicted MRIs and a combination of real and predicted MRIs for TP53, PIK3CA and CDH1 with testing set containing 20 percent of total

Gene (# of mutated patients/total patients)	ROC AUC	PR AUC
TP53 multi-omic logistic regression (235/690)	0.9400	0.9009
TP53 with real MRI (21/50)	1.0000	1.0000
TP53 with cGAN MRI (235/690)	0.9477	0.8926
TP53 with cGAN + real MRI (256/740) PIK3CA multi-omic logistic regression(247/690) PIK3CA with real MRI (11/50) PIK3CA with cGAN MRI (247/690) PIK3CA with cGAN + real MRI (258/740)	**0.9508** 0.7209 0.8333 0.7407 **0.7515**	**0.9301** 0.5413 0.8110 0.6360 **0.7184**
CDH1 multi-omic logistic regression (112/690)	0.8068	0.4342
CDH1 with real MRI (11/50)	0.9167	0.9083
CDH1 with cGAN MRI (112/690)	0.8105	0.4861
CDH1 with cGAN + real MRI (123/740)	**0.8136**	**0.5007**

### Performance of cGAN MRIs in BC subtype prediction

The performance of the cGAN generated MRIs for BC subtype prediction was assessed using two different methods: a multi-class CNN and an XGBoost classifier utilizing features extracted through Pyradiomics [44]. Notably, the dataset comprising of cGAN and real MRIs demonstrated the highest performance across all three metrics (Table 5). While the performance of Pyradiomics is comparatively lower than that of the CNN, a similar trend is evident, where the combined dataset out scored the cGAN dataset in all three metrics.

**Table 5:** Precision, Recall, and F1 scores of subtype multiclass classification task using CNN and Pyradiomics. Models were trained with cGAN MRIs, and a combined dataset of cGAN and real patient MRIs. 20 percent testing sets were used for evaluation

Model (Dataset)	Precision	Recall	F1
**CNN** (cGAN MRI)	0.7988	0.7917	0.7695
**CNN** (cGAN cGAN + real MRI)	0.8444	0.8435	0.8336
**PyRadiomics (cGAN MRI)** **PyRadiomics** (cGAN + real MRI)	0.4879 0.5001	0.5507 0.5732	0.4386 0.5344

## Discussion

The present study showcased the potential of cGANs for synthetic MRI generation in BC patients. The quantitative difference between the baseline models and the cGAN-generated MRIs as indicated by the FID score, solidifies cGANs as a promising tool for BC MRI generation. The predictive power of these synthetically generated images establishes this method as a powerful alternative to the costly and invasive nature of current BC diagnosis methods. However, further refinement is required to improve the performance of the current model. The dataset used for training of the cGAN was extremely limited, consisting of only 50 MRIs from 28 patients. To augment this limited dataset, multiple MRIs obtained from the same patient were treated as separate cases.

As observed from the loss curve depicted in Additional file 1: Fig. S3, the cGAN model demonstrates stabilization towards 0.25 for the discriminator and 0.45 for the generator, indicating a balanced state between the two networks. Notably, while fluctuations in the loss values were observed during the training stage in the loss curve, the generated images using the well-trained model exhibited a high level of consistency and quality both visually and quantitively. While the fluctuations during training may raise concerns about the model’s convergence, it is important to emphasize that ultimately, it is the quality of the predicted MRIs that are of importance. As a result, the consistent construction of MRIs using solely multi-omic data in the prediction stage indicates that the model has effectively learned and generalized from the training data, producing reliable predictions with satisfactory image quality.

The quality of the MRIs produced by the well-trained cGAN model has been further demonstrated by its application in training a CNN for predicting mutation status. In the present study, we selected the *TP53, PIK3CA*, and *CDH1* genes due to their prognostic values and high occurrence of mutations in the dataset. *TP53* is a tumor suppressor gene that regulates DNA repair mechanisms and apoptosis. The number of mutated patients from the TCGA dataset 235 out of 690 which is roughly 34 percent and is consistent with frequencies observed from previous literature [45]. Mutated versions of TP53 can be detrimental or beneficial to clinical outcome based on the treatment provided and therefore, its mutation status is of great benefit when curating a treatment plan. PIK3CA mutations occurs in approximately 36 percent of all BC patients, which aligns with the 36 percent mutation rate as observed in our dataset. The gene encodes for the catalytic subunit of the phosphatidylinositol 3-kinase complex known as, p110α [46]. Preliminary studies have demonstrated that hyperactivation of this pathway may confer resistance to both HER2 and endocrine therapies [47–49]. CDH1 is a member of the cadherins superfamily, which are calcium-dependent adhesion molecules that partake in cell recognition, tissue morphogenesis and tumor suppression. 112 patients in the present dataset are mutated for this gene and accounts for around 16 percent of the total.

The CNN was designed to perform binary classifications on the aforementioned genes of interest, providing important insights into the breast tumor heterogeneity. Moreover, the CNN serves as a validation method for the quality of the cGAN-generated MRIs. It is crucial that the underlying multi-omic data of each patient is accurately transferred and extractable from the image to enable potential prognostic use of the generated MRIs. Poor CNN performance from the cGAN MRIs and superior performance when trained using real patient data would indicate that although the cGAN generated MRIs are visually pleasing, it lacks the hidden features that can be utilized for mutation status prediction and BC prognosis. Early detection of these biomarker mutation status is crucial for the success of a treatment plan, given the significant impact these driving genes have on BC progression.

The CNN achieved an impressive AUC score of 1.0000 for both ROC and PR when trained using real patient MRIs, indicating its ability to extract crucial features for accurately classifying the mutation status of TP53. Notably, when the CNN was trained using the joint dataset with predicted MRIs generated by the cGAN and real patient data, AUC values exceeding 0.90 were obtained for both metrics. The high scores affirm the quality and predictive power of the cGAN—produced MRIs. However, it is important to recall that the well-trained cGAN’s MRIs received an FID score of 1.31, indicating room for improvement and the potential absence of important features. This discrepancy is reflected in the lower AUC scores obtained by the CNN trained with cGAN images. The distance of 1.31 and the difference between the real and generated images may have hindered the ability of cGAN MRIs to achieve a perfect 1.0000 AUC score for mutation status prediction. This trend can be observed in the other genes of interest, PIK3CA and CDH1, where the real images produce very high AUC scores while the joint CNN trails behind in terms of predictive power. The AUC for the real image CNN achieves > 0.80 for both ROC and PR while the combined CNN scores 0.7515 and 0.7184, respectively, for PIK3CA. This suggests that the real patient MRI had already lacked features that are important to perform highly accurate predictions. With the cGAN being trained on these images, the produced MRIs will follow similar patterns and lead to poorer performance. This is, however, not the case for CDH1. This gene achieved > 0.90 AUC values for real CNN while the PR-AUC for the combined CNN was only 0.5007. This vast difference in performance can be attributed to the missing features present on the cGAN produced MRIs and not an issue with the real patient MRIs. To further examine the clinical applicability of theses artificial MRIs, we evaluated its ability to be used in BC subtype classification. The combined dataset was able to achieve precision, recall and F1 scores of > 0.80 while the cGAN dataset scored just below 0.80.

Overall, the high AUC scores obtained by the CNN trained with a joint dataset comprising of both real and cGAN generated artificial MRIs provide further evidence for the feasibility of utilizing cGANs for the synthesis of clinically viable BC MRIs and can be used as a low–cost, non-invasive alternative to current methods. It can be noted that the dataset incorporating both real and synthetic MRIs, outperformed the predictions using pure multi-omic data for mutation status prediction and cGAN dataset for subtype classification. This observation reinforces the utility of the cGAN as an imaging modality with the ability to enhance predictive accuracy for the mutation status of these key BC—driving genes and BC subtypes. The images exhibit good visual presentation and include many hidden features that can be extracted for predicting mutation status of various genes, particularly TP53 and PIK3CA. However, it is crucial to acknowledge the limitations of the cGAN model due to the limited training dataset of 50 MRIs. The high heterogeneity of BC poses a major challenge for this small training sample as there is great genetic and phenotypic diversity among patients. As evident in cGAN MRIs, the small dataset hinders the extraction of complex features and reduces its generalizability to a wider BC patient population. Future work could investigate the performance of the model when trained on the top-down BC images or to employ data augmentation techniques to expand the training dataset. Expanding the training dataset by acquiring more patient data would likely enhance the model’s generalization capabilities and potentially improve the overall quality of the generated MRIs. Consequently, the predictive abilities of the CNN maybe improved as well. With continued enhancements, the cGAN generated MRIs could provide valuable clinical insights for patients by predicting the mutation status of key BC driving genes and classifying the disease as the correct subtype. This method presents a cost effective and time-efficient alternative compared to traditional methods like genetic testing.

## Conclusions

This study lays the foundation for future BC related machine learning studies by solidifying cGAN as a potential tool for synthetic BC MRI generation. The cGAN-based augmentation of the existing BC database offers a solution to ethical and privacy concerns associated with using patient data for research purposes. Importantly, our findings also suggest that cGAN-generated MRIs could be used to estimate the patients BC subtype and mutation status of BC-driving genes and can be useful for the construction of personalized treatment plans and aid in BC prognosis. If future improvements for generalizability and survival analysis are conducted, this method could serve as a non-invasive and cost-effective alternative to invasive biopsy procedures, enabling early detection of BC. Therefore, this novel approach could prove to be of great significance in the field of radiogenomic research, with potential for widespread clinical application in the future.

### Supplementary Information

**Additional file 1: Figure S1.** Top-down MRI view. Full 32 slices of top-down MRI view shown in Fig. 1. **Figure S2.** Side MRI view. Full 32 slices of side MRI view shown in Fig. 1. **Figure S3.** cGAN loss curves. The loss curves of the cGAN trained for 1200 epochs using the mean squared error loss function. Blue curve depicts the generator loss while the orange curve represents the loss for the discriminator. **Figure S4.** Real MRI for the patient TCGA-AO-A12E. Full 32 slices of the real patient MRI shown in panel A of Fig. 3. **Figure S5.** cGAN generated MRI for the patient TCGA-AO-A12E. Full 32 slices of the cGAN generated MRI shown in panel B of Fig. 3. **Figure S6.** Resnet 18 autoencoder generated MRI for the patient TCGA-AO-A12E. Full 32 slices of the Resnet 18 autoencoder generated MRI shown in **C** of Fig. 3. **Figure S7.** Traditional autoencoder generated MRI for the patient TCGA-AO-A12E. Full 32 slices of the autoencoder generated MRI shown in **D** of Fig. 3. **Figure S8.** CNN MSE loss curve, ROC and PR curves for *PIK3CA*. Top panel depicts CNN trained using real patient MRIs, middle panel represents CNN trained on cGAN predicted MRIs, and bottom panel for CNN trained with both real and cGAN generated MRIs. **Figure S9.** CNN MSE loss curve, ROC and PR curves for *CDH1*. Top panel depicts CNN trained using real patient MRIs, middle panel represents CNN trained on cGAN predicted MRIs, and bottom panel for CNN trained with both real and cGAN generated MRIs. **Figure S10.** ROC AUC and PR AUC for the chosen genes. Logistic regression with L1 regularization was trained to predict the mutation status of the 3 chosen genes. ROC AUC and PR AUC were calculated and plotted **A** TP53, **B** PIK3CA, **C** CDH1. **Table S1.** ROC AUC and PR AUC scores of CNN trained with cGAN predicted images for TP53, PIK3CA and CDH1 with various portions of the testing set. **Table S2.** CLAIMs checklist for artificial intelligence in medical imaging.

## Data Availability

The datasets used for analysis during the current study are available from the TCGA archives (https://www.cancer.gov/tcga) and TCIA (https://www.cancerimagingarchive.net).
