# Supplementary material for: Conditional generative adversarial network driven radiomic prediction of mutation status based on magnetic resonance imaging of breast cancer
Source: J Transl Med. 2024 Mar 2;22:226. doi: 10.1186/s12967-024-05018-9 (PMC10908206; doi:10.1186/s12967-024-05018-9)
Supplement: Supplementary file 1 — Additional file 1: Figure S1. Top-down MRI view. Full 32 slices of top-down MRI view shown in Fig. 1. Figure S2. Side MRI view. Full 32 slices of side MRI view shown in Fig. 1. Figure S3. cGAN loss curves. The loss curves of the cGAN trained for 1200 epochs using the mean squared error loss function. Blue curve depicts the generator loss while the orange curve represents the loss for the discriminator. Figure S4. Real MRI for the patient TCGA-AO-A12E. Full 32 slices of the real patient MRI shown in panel A of Fig. 3. Figure S5. cGAN generated MRI for the patient TCGA-AO-A12E. Full 32 slices of the cGAN generated MRI shown in panel B of Fig. 3. Figure S6. Resnet 18 autoencoder generated MRI for the patient TCGA-AO-A12E. Full 32 slices of the Resnet 18 autoencoder generated MRI shown in C of Fig. 3. Figure S7. Traditional autoencoder generated MRI for the patient TCGA-AO-A12E. Full 32 slices of the autoencoder generated MRI shown in D of Fig. 3. Figure S8. CNN MSE loss curve, ROC and PR curves for PIK3CA. Top panel depicts CNN trained using real patient MRIs, middle panel represents CNN trained on cGAN predicted MRIs, and bottom panel for CNN trained with both real and cGAN generated MRIs. Figure S9. CNN MSE loss curve, ROC and PR curves for CDH1. Top panel depicts CNN trained using real patient MRIs, middle panel represents CNN trained on cGAN predicted MRIs, and bottom panel for CNN trained with both real and cGAN generated MRIs. Figure S10. ROC AUC and PR AUC for the chosen genes. Logistic regression with L1 regularization was trained to predict the mutation status of the 3 chosen genes. ROC AUC and PR AUC were calculated and plotted A TP53, B PIK3CA, C CDH1. Table S1. ROC AUC and PR AUC scores of CNN trained with cGAN predicted images for TP53, PIK3CA and CDH1 with various portions of the testing set. Table S2. CLAIMs checklist for artificial intelligence in medical imaging. [file 12967_2024_5018_MOESM1_ESM.pdf]

Additional Files

Figures

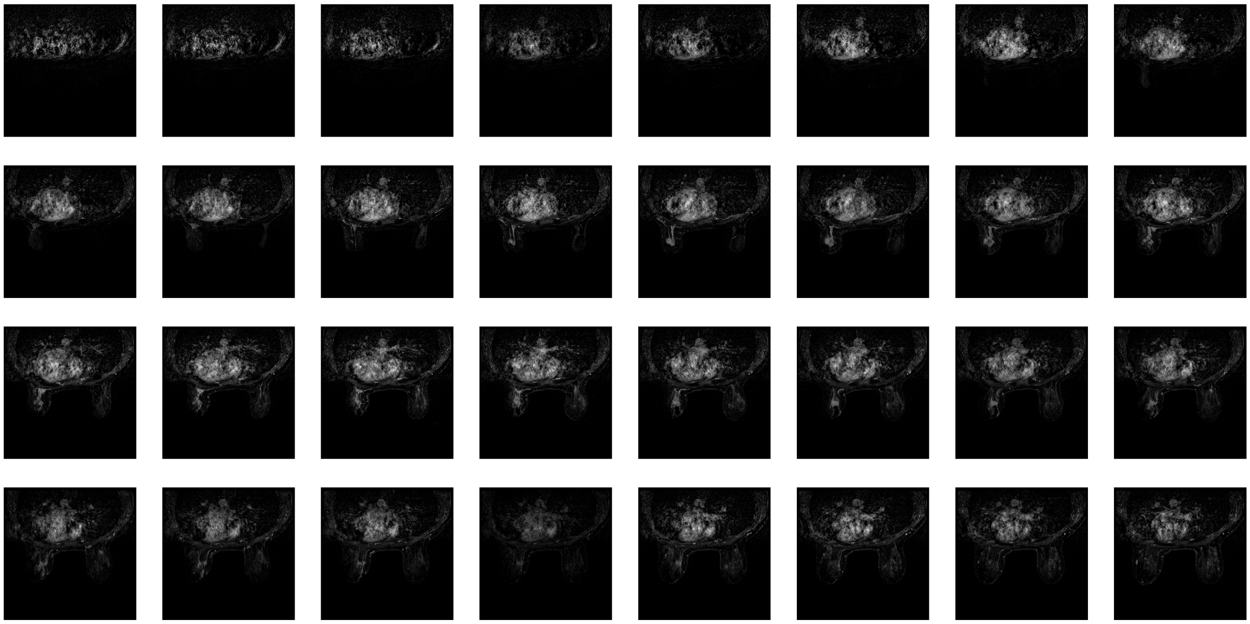

Figure S1. Top-down MRI view. Full 32 slices of top-down MRI view shown in Fig. 1.

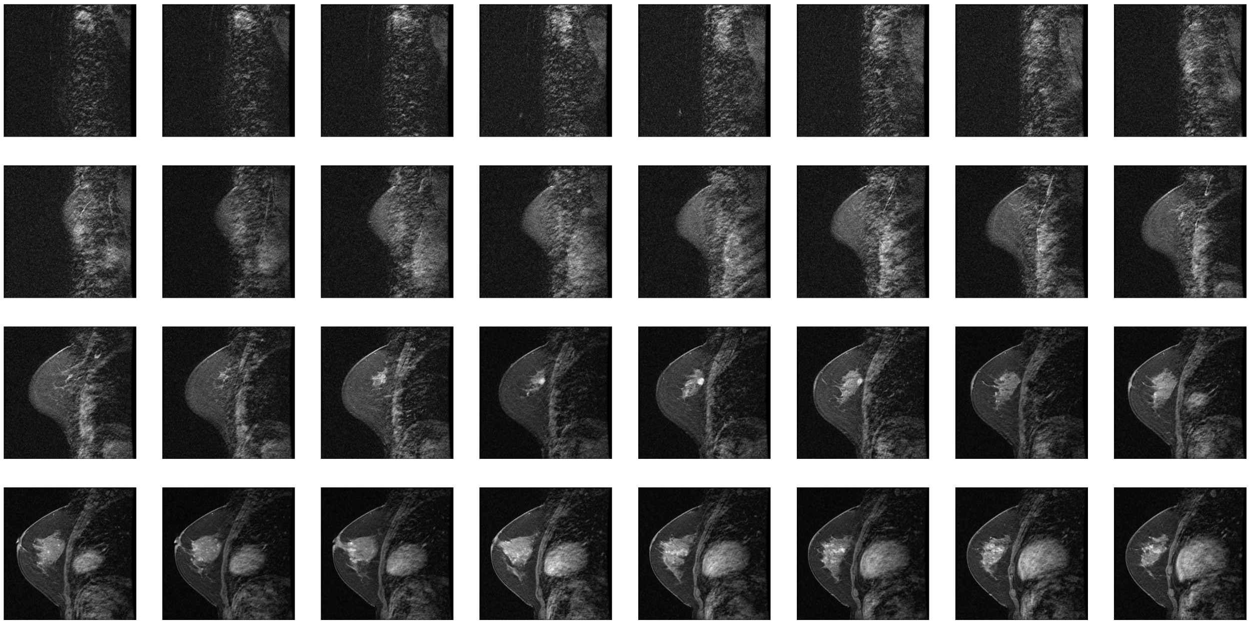

Figure S2. Side MRI view. Full 32 slices of side MRI view shown in Fig. 1

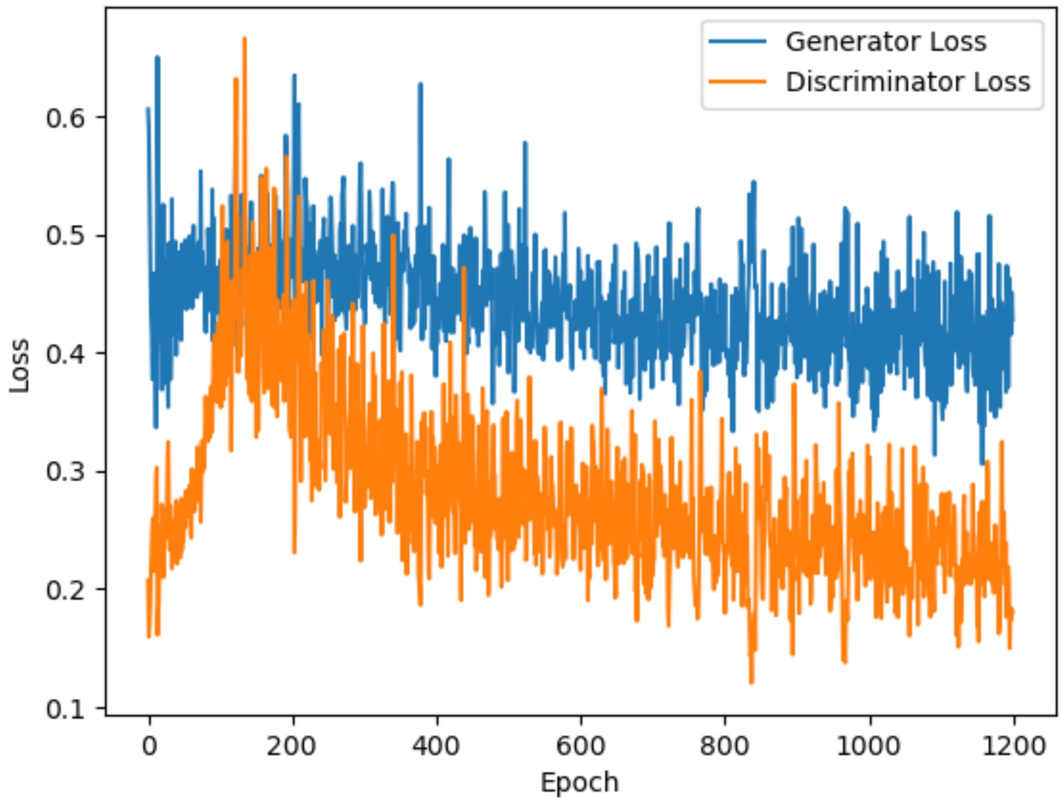

Figure S3. cGAN loss curve. The loss curve of the cGAN trained for 1200 epochs using the mean squared error loss function. Blue line depicts the generator loss while the orange line represents the loss for the discriminator.

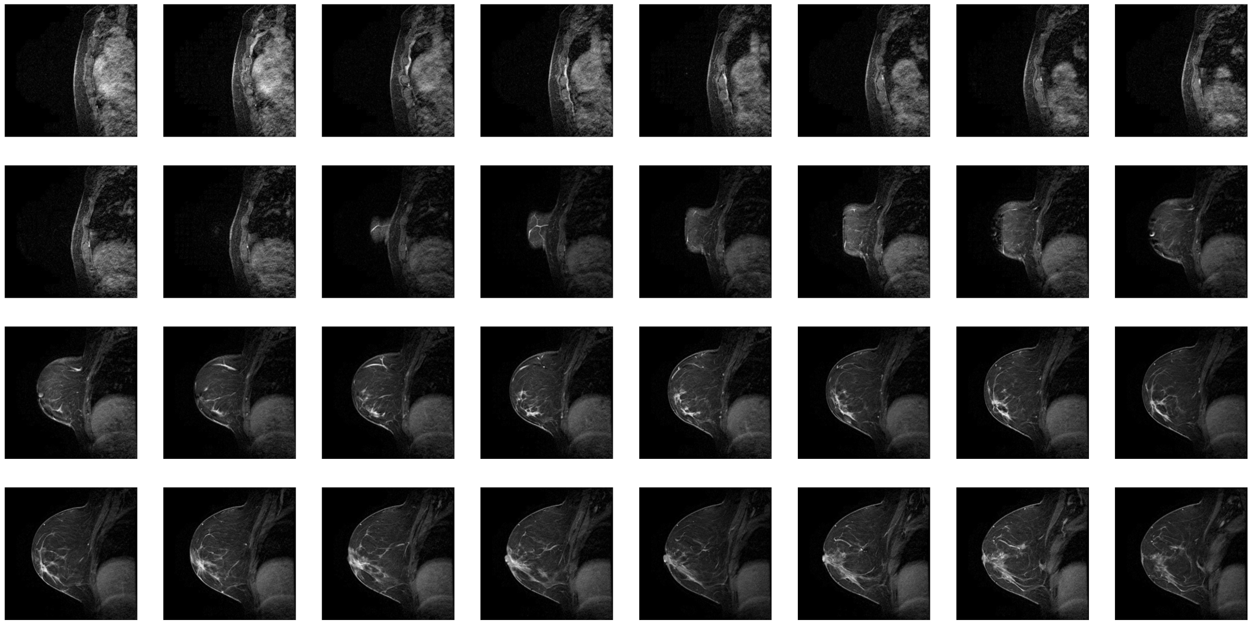

Figure S4. Real patient MRI for patient TCGA-AO-A12E. Full 32 slices of the real patient MRI shown in panel A of Fig. 3.

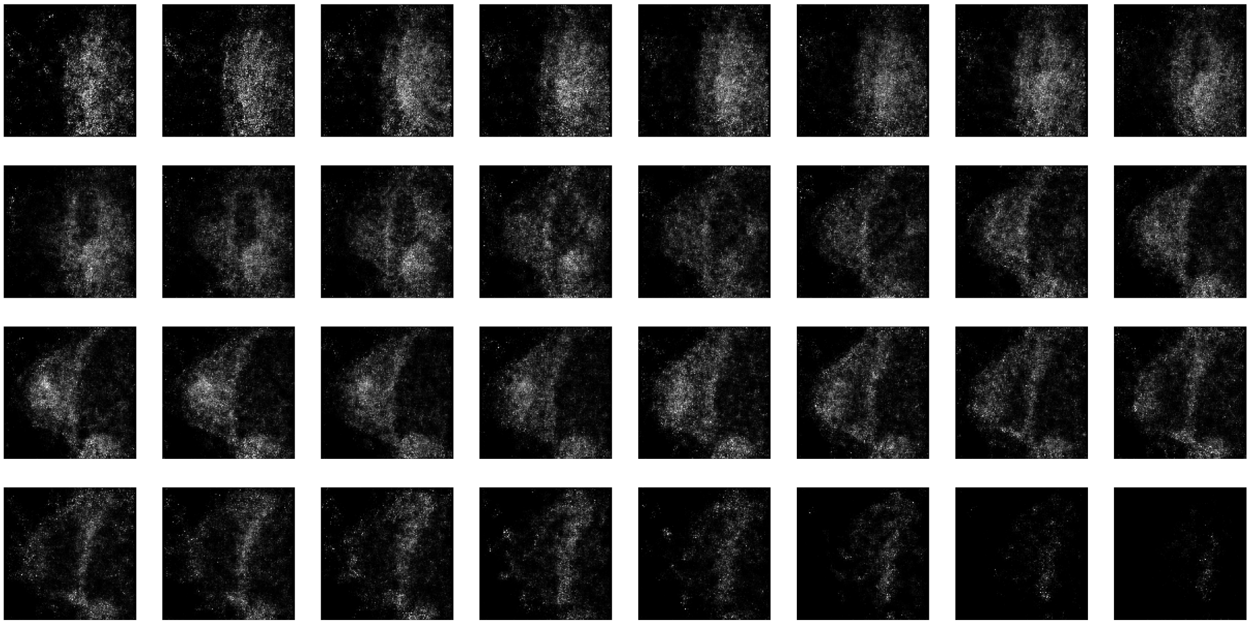

Figure S5. cGAN generated MRI for patient TCGA-AO-A12E. Full 32 slices of the cGAN generated MRI shown in panel B of Fig. 3.

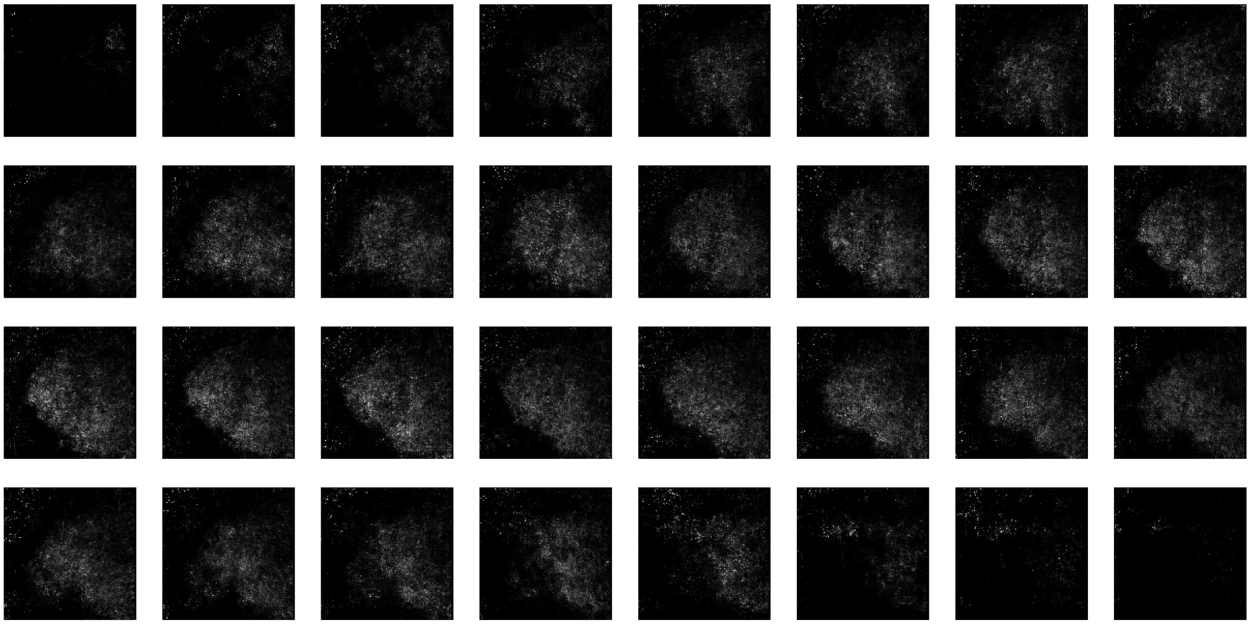

Figure S6. Resnet 18 autoencoder generated MRI for patient TCGA-AO-A12E. Full 32 slices of the Resnet 18 autoencoder generated MRI shown in panel C of Fig. 3.

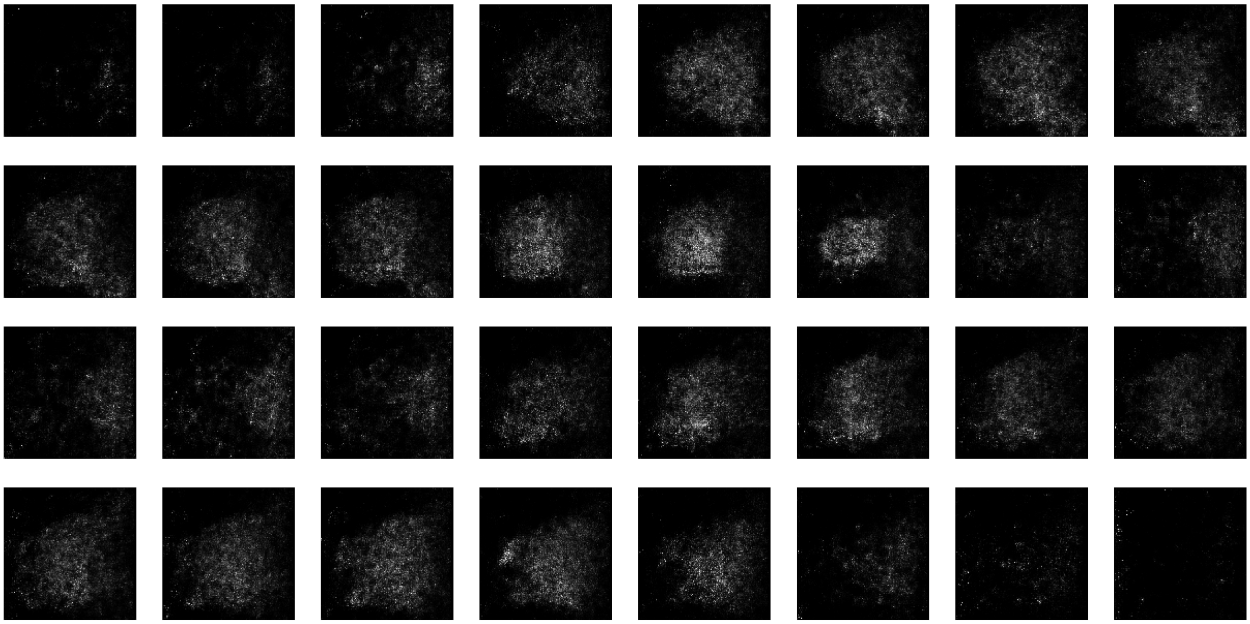

Figure S7. Traditional autoencoder generated MRI for patient TCGA-AO-A12E. Full 32 slices of the autoencoder generated MRI shown in panel D of Fig. 3.

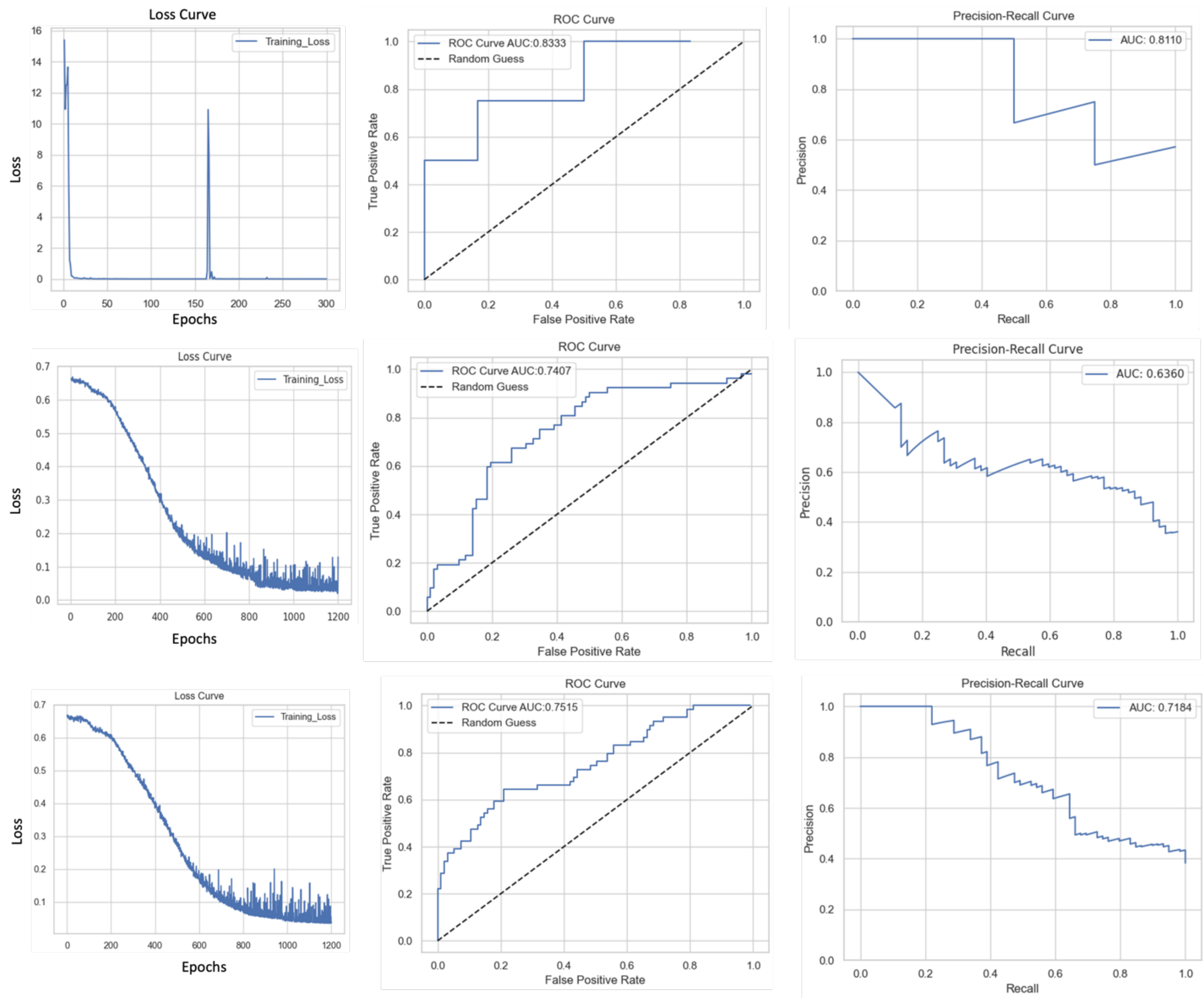

Figure S8. CNN BCE loss curve, ROC and PR curves for *PIK3CA*. Top panel depicts CNN trained using real patient MRIs, middle panel represents CNN trained on cGAN predicted MRIs, and bottom panel for CNN trained with both real and cGAN generated MRIs.

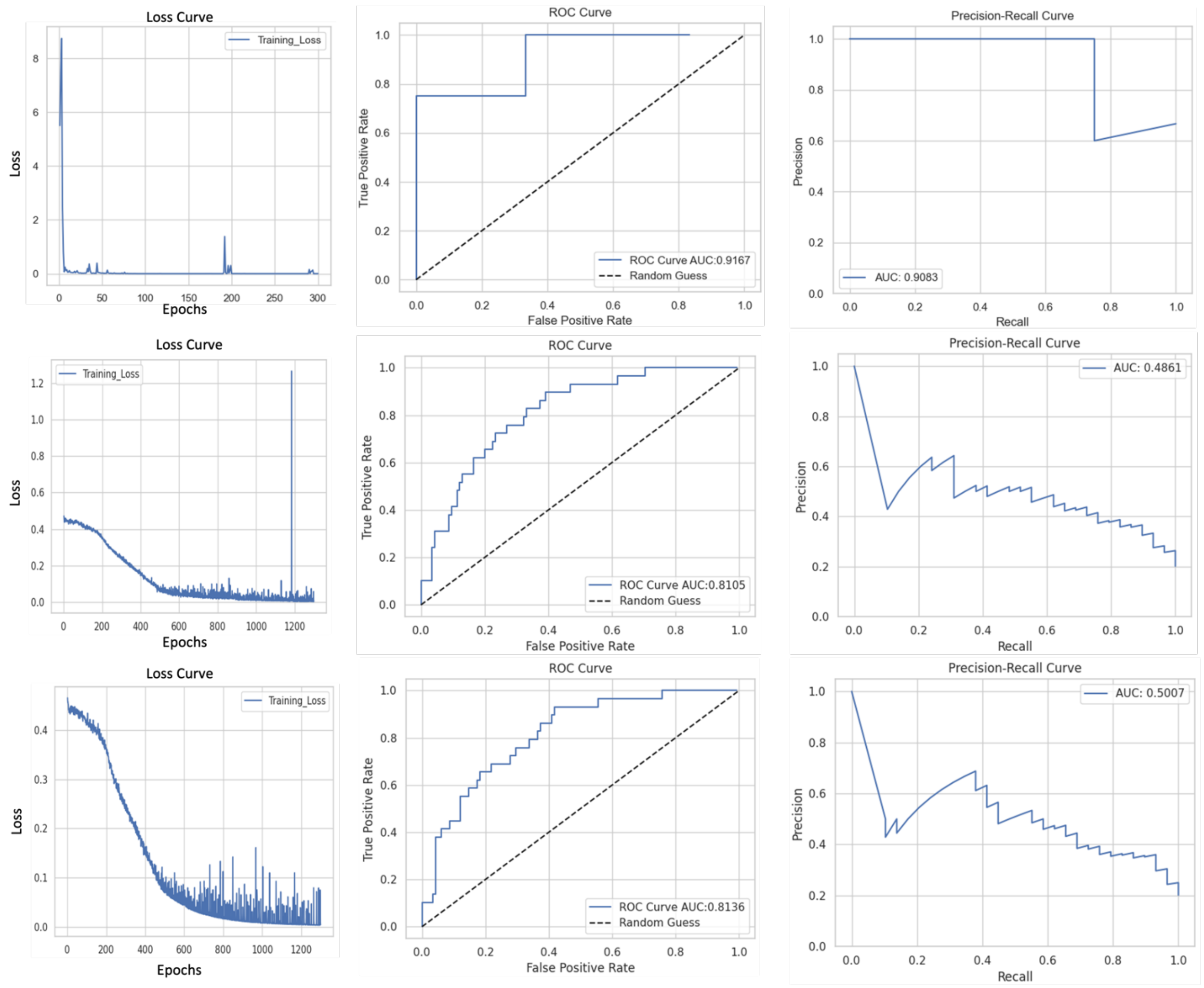

Figure S9. CNN BCE loss curve, ROC and PR curves for *CDH1*. Top panel depicts CNN trained using real patient MRIs, middle panel represents CNN trained on cGAN predicted MRIs, and bottom panel for CNN trained with both real and cGAN generated MRIs.

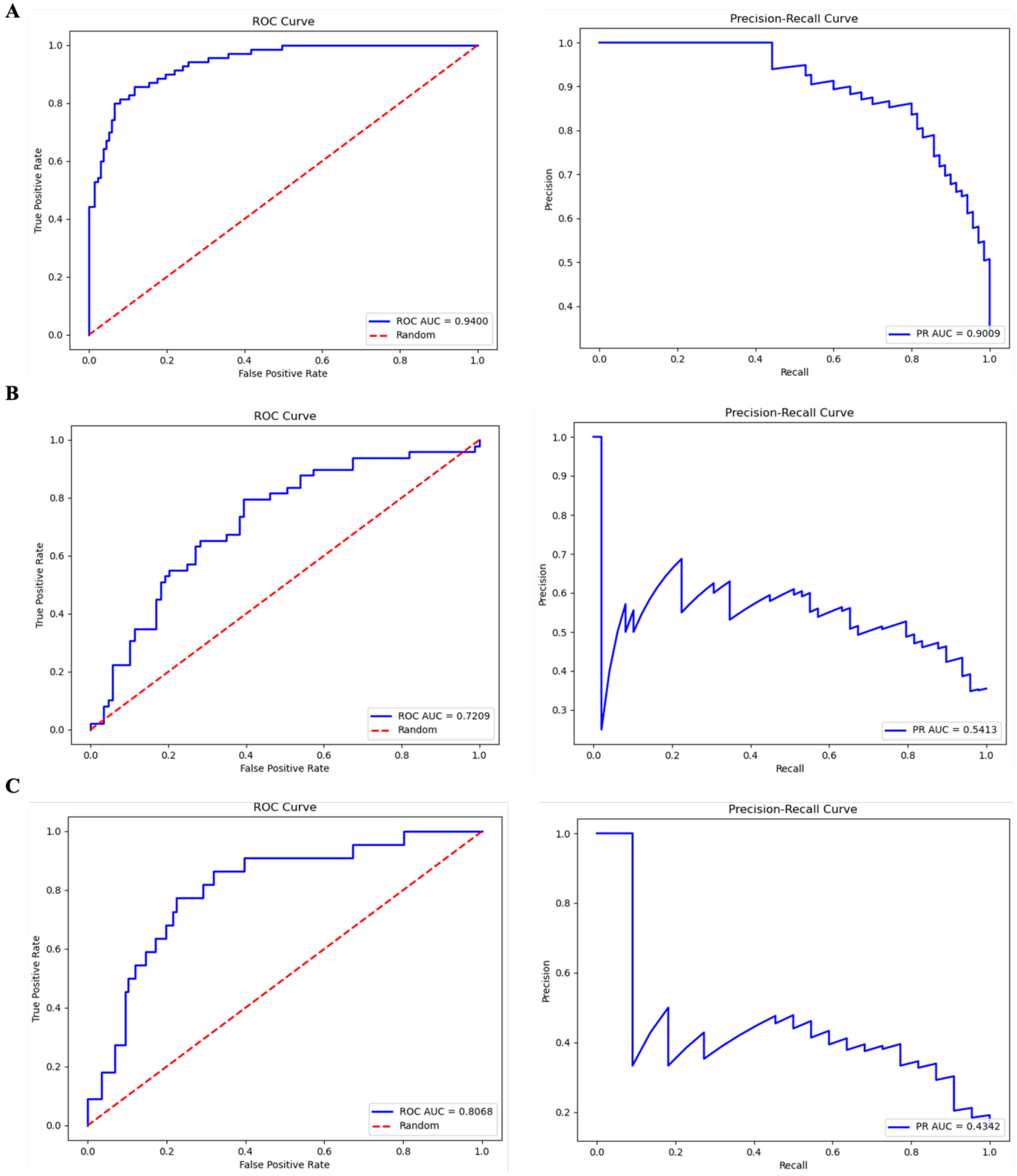

Figure S10. ROC AUC and PR AUC for chosen genes. Logistic regression with L1 regularization was trained to predict the mutation status of the 3 chosen genes. ROC AUC and PR AUC were calculated and plotted A) TP53 B) PIK3CA C) CDH1

Tables

Table S1. ROC AUC and PR AUC scores of CNN trained with cGAN predicted images for TP53, PIK3CA and CDH1 with various portions of the testing set.

Gene (# of mutated patients / total patients)	ROC AUC	PR AUC
**TP53** with cGAN MRI (235/690) – 10%	0.9231	0.8418
**TP53** with cGAN MRI (235/690) – 20%	0.9477	0.9261
**TP53** with cGAN MRI (235/690) – 30%	0.8834	0.8271
**PIK3CA** with cGAN MRI (247/690) – 10%	0.7400	0.4811
**PIK3CA** with cGAN MRI (247/690) – 20%	0.7407	0.6360
**PIK3CA** with cGAN MRI (247/690) – 30%	0.7358	0.6269
**CDH1** with cGAN MRI (112/690) – 10%	0.8542	0.5455
**CDH1** with cGAN MRI (112/690) – 20%	0.8105	0.4861
**CDH1** with cGAN MRI (112/690) – 30%	0.7938	0.3537

Table S2. CLAIMs checklist for artificial intelligence in medical imaging

Section / Topic	No.	Item	
TITLE / ABSTRACT			
	**1**	Identification as a study of AI methodology, specifying the category of technology used (e.g., deep learning)	✓
	**2**	Structured summary of study design, methods, results, and conclusions	✓
INTRODUCTION			
	**3**	Scientific and clinical background, including the intended use and clinical role of the AI approach	✓
	**4**	Study objectives and hypotheses	✓
METHODS			
Study Design	**5**	Prospective or retrospective study	✓
	**6**	Study goal, such as model creation, exploratory study, feasibility study, non-inferiority trial	✓
Data	**7**	Data sources	✓
	**8**	Eligibility criteria: how, where, and when potentially eligible participants or studies were identified (e.g., symptoms, results from previous tests, inclusion in registry, patient-care setting, location, dates)	**N/A**
	**9**	Data pre-processing steps	✓
	**10**	Selection of data subsets, if applicable	✓

	**11**	Definitions of data elements, with references to Common Data Elements	**N/A**
	**12**	De-identification methods	**N/A**
	**13**	How missing data were handled	**N/A**
Ground Truth	**14**	Definition of ground truth reference standard, in sufficient detail to allow replication	✓
	**15**	Rationale for choosing the reference standard (if alternatives exist)	✓
	**16**	Source of ground-truth annotations; qualifications and preparation of annotators	✓
	**17**	Annotation tools	**N/A**
	**18**	Measurement of inter- and intrarater variability; methods to mitigate variability and/or resolve discrepancies	**N/A**
Data Partitions	**19**	Intended sample size and how it was determined	**N/A**
	**20**	How data were assigned to partitions; specify proportions	**N/A**
	**21**	Level at which partitions are disjoint (e.g., image, study, patient, institution)	
Model	**22**	Detailed description of model, including inputs, outputs, all intermediate layers and connections	✓
	**23**	Software libraries, frameworks, and packages	✓
	**24**	Initialization of model parameters (e.g., randomization, transfer learning)	✓
Training	**25**	Details of training approach, including data augmentation, hyperparameters, number of models trained	✓
	**26**	Method of selecting the final model	✓
	**27**	Ensembling techniques, if applicable	**N/A**
Evaluation	**28**	Metrics of model performance	✓
	**29**	Statistical measures of significance and uncertainty (e.g., confidence intervals)	✓
	**30**	Robustness or sensitivity analysis	✓
	**31**	Methods for explainability or interpretability (e.g., saliency maps), and how they were validated	✓
	**32**	Validation or testing on external data	✓
RESULTS			
Data	**33**	Flow of participants or cases, using a diagram to indicate inclusion and exclusion	✓
	**34**	Demographic and clinical characteristics of cases in each partition	**N/A**
Model performance	**35**	Performance metrics for optimal model(s) on all data partitions	✓

	**36**	Estimates of diagnostic accuracy and their precision (such as 95% confidence intervals)	**N/A**
	**37**	Failure analysis of incorrectly classified cases	**N/A**
DISCUSSION			
	**38**	Study limitations, including potential bias, statistical uncertainty, and generalizability	✓
	**39**	Implications for practice, including the intended use and/or clinical role	✓
OTHER INFORMATION			
	**40**	Registration number and name of registry	**N/A**
	**41**	Where the full study protocol can be accessed	✓
	**42**	Sources of funding and other support; role of funders	✓
